# Meckel Gruber Syndrome: Second trimester diagnosis of a case in a non-consanguineous marriage

**DOI:** 10.12669/pjms.291.2930

**Published:** 2013

**Authors:** Areej Alam, Mehreen Adhi, Raffat Bano, Aisha Zubair, Ammara Mushtaq

**Affiliations:** 1 Areej Alam, Dow Medical College, Dow University of Health Sciences, Karachi, Pakistan.; 2 Mehreen Adhi, Department of Ophthalmology, Tufts University School of Medicine, Boston, USA.; 3 Raffat Bano, Department of Obs & Gyn, Aga Khan Hospital for Women, Karimabad, Karachi, Pakistan.; 4 Aisha Zubair, Dow Medical College, Dow University of Health Sciences, Karachi, Pakistan.; 5 Ammara Mushtaq, Dow Medical College, Dow University of Health Sciences, Karachi, Pakistan.

**Keywords:** Meckel Gruber Syndrome, Dysencephalia Splanchnicocystica

## Abstract

Meckel-Gruber Syndrome (MKS) is a rare, autosomal recessive genetic disorder, incompatible with life. It is characterized by enlarged polycystic kidneys and post axial polydactyly. Foetal or neonatal death is caused by pulmonary hypoplasia. We report a case of a 35 year old woman who presented at 7 weeks of gestation of her sixth pregnancy. A transabdominal anomaly ultrasound performed for her current pregnancy at 18 weeks of gestation showed features consistent with MKS. The termination of pregnancy was declined and a live newborn female was delivered via an emergency caeserean section at 34 weeks of gestation due to previous history of lower segment caesarean section (LSCS) & leaking. Physical examination of the neonate confirmed the features of MKS. The neonate died within 4-5 hours of birth. This case represented a second trimester diagnosis of a recurrent case of MKS in a non-consanguineous marriage.

## Introduction

 Meckel-Gruber Syndrome (MKS) also known as Dysencephalia Splanchnicocystica, was first described by J. R. Meckel in 1822. It is an autosomal recessive genetic disease and occurs in all races and ethnicities. The highest incidence is reported in Finnish population of 1 in 9000.^1^ To date, up to 200 cases of MKS have been reported in the literature.^2^

 MKS is a rare disease with a recurrence rate of 1 in 4 (25%).^3^ It has been suggested to be caused by failure of mesodermal induction. The typical triad of MKS involves meningo-encephalocoele, enlarged polycystic kidneys and post axial polydactyly. Numerous other anomalies associated with MKS include liver dysfunction, cleft palate, cardio-vascular diseases, oligohydramnios, genital deformation, bowed legs, microcephaly and hydrocephalus.^1^^,^^2^

 We report a case of MKS diagnosed at 18 weeks of gestation in a foetus carried and delivered by a female having a previous history MKS.

## Case Reports

 A 35 year old pregnant female presented at the out-patients’ clinic in a secondary care hospital, the Aga Khan Hospital for Women in Karachi, Pakistan at 7th week of gestation. She had a history of her first pregnancy resulting in an unexplained intrauterine death (IUD) at term, accompanied by post-partum haemorrhage (PPH), and her second pregnancy ending in still birth due to MKS, after an elective caesarean section. This was followed by three pregnancies ending in first trimester miscarriages after which a workup for recurrent miscarriages performed was reported to be negative. The current pregnancy was her sixth. She was an otherwise healthy female living with her husband and two adopted children, and her marriage was non-consanguineous.

 A trans-abdominal anomaly ultrasound was performed at 18 weeks of gestation which showed features consistent with MKS. This included a small and irregular foetal skull suggestive of microcephaly and a defect in occipital region suggestive of posterior encephalocoele. A large cystic structure with a spoke wheel pattern was noted in the region of the neck, suggesting a cystic hygroma. The heart was deformed and the interventricular septum could not be visualized. Mild pericardial effusion was noted and the kidneys were enlarged and echogenic suggesting an infantile form of polycystic kidney disease. Both femurs appeared bent. These features were suggestive of the diagnosis of MKS. The patient was referred to a Paediatric Surgeon, who reviewed the ultrasonography findings.

 The patient was counselled regarding the lethal outcome of MKS. However, she decided to continue the pregnancy. Further genetic and family planning counselling was offered after which she requested for tubal ligation at the termination of her current pregnancy. She was a non-compliant patient and lost follow up for 10 weeks and later presented in emergency at 34th week of gestation with leaking and labour pains. An emergency C-section was performed due to a breech presentation, previous LSCS and leaking per vagina, followed by tubal ligation.

 A live female baby weighing 1.2 kg was delivered and examined by an experienced Paediatric Surgeon. The diagnosis of MKS was confirmed based on the clinical features and the ultrasonography findings. The neonate died 4-5 hours after birth.

## Discussion

 MKS is a rare autosomal recessive genetic disorder, usually diagnosed on ultrasonography in the second trimester. The mean gestational age at diagnosis is 19 weeks.^4^ However, some reports have suggested an early diagnosis (in the first trimester) may be possible and preferable.^5^^,^^6^ MKS is an invariably fatal disorder, however Gazioglu et al. reported an unusual case of MKS with a good outcome initially with appropriate treatment and the infant dying at 7 months of age.^3^ MKS affects both genders equally^7^, and consanguinity has been reported to be an important factor in the genetic basis of the disease^3^^,^^8^ and accordingly, its prevalence may be higher in Pakistan as about two-thirds of the marriages in Pakistan are consanguineous.^8^ A few cases of MKS have been reported from Pakistan previously.^8^^,^^9^

 The typical presentation of MKS involves the clinical triad of meningo-encephalocoele, enlarged polycystic kidneys and post axial polydactyly. For a definitive diagnosis of MKS, two of the three major anomalies should be present.^3^ The most striking anomaly in MKS is occipital encephalocoele.^9^ Other cranio-facial abnormalities may also be present.^10^ Enlarged polycystic kidney or cystic dysplasia is the most constant feature of MKS.^10^^,^^11^ The kidneys may be enlarged up to 10-20 times normal^10^, leading to oligohydramnios and pulmonary hypoplasia; the most common cause of death in MKS. Postaxial polydactyly is the most variable of the three major presentations of MKS^10^, and when present, affects all four limbs. Hepatic lesions are consistent findings on post-mortem examination.^10^

 The diagnosis of MKS is confirmed by different methods in the first, second or third trimester of pregnancy or by the physical examination of the neonate after birth. However, prenatal ultrasonography is currently the most reliable method for the diagnosis of MKS.^12^ In the second trimester, severe oligohydramnios may impair the visualization of the physical defects.^7^^,^^11^ Alpha-fetoprotein (AFP) levels rise in maternal during 11-16 weeks of gestation in such cases^3^^,^^11^ but it was not measured in our case. Chromosome analysis by amniocentesis or chorionic villous sampling is also an important diagnostic method.^10^

 Several gene loci have been suggested for the mapping of this rare genetic disease, which might explain the clinical heterogeneity of the disease.^4^ Chromosomal analysis has revealed chormosome 17 to be linked to MKS.^10^ The mortality of MKS is 100% with IUD or death soon after birth.^10^^,^^11^

## Conclusions

 In conclusion, we have presented a case of MKS, in which the diagnosis was made at 18 weeks of gestation based on ultrasonographic findings of the typical triad of the disease. These were further confirmed by the physical examination of the neonate after birth. The newborn female with MKS was born to a non-consanguineous marriage.

## References

[1] Ahdab-Barmada M, Claassen D (1990). A distinctive triad of malformations of the central nervous system in the Meckel-Gruber syndrome. J Neuropathol Exp Neurol.

[2] Carter, SM (2002). Meckel-Gruber syndrome. eMedicine. http://emedicine.medscape.com/article/946672-overview.

[3] Gazioglu N, Vural M, Seckin MS, Tuysuz B, Akpir E, Kuday C (1998). Meckel-Gruber syndrome. Childs Nerv Syst.

[4] Ickowicz V, Eurin D, Maugey-Laulom B, Didier F, Garel C, Gubler MC (2006). Meckel-Gruber syndrome: sonography and pathology. Ultrasound Obstet Gynecol.

[5] Braithwaite JM, Economides DL (1995). First-trimester diagnosis of Meckel-Gruber syndrome by transabdominal sonography in a low-risk case. Prenat Diagn.

[6] Mazneŭkova V, Kamenov E, Dimitrova L (2002). Ultrasound diagnosis of Meckel-Gruber syndrome at 13 weeks of gestation in families at risk-a case report and literature review. Akush Ginekol (Sofiia).

[7] Alexiev BA, Lin X, Sun CC, Brenner DS (2006). Meckel-Gruber syndrome: pathologic manifestations, minimal diagnostic criteria, and differential diagnosis. Arch Pathol Lab Med.

[8] Munim S, Nadeem S, Sikandar R (2004). First trimester diagnosis of Meckel Gruber syndrome in pregnancy. J Pak Med Assoc.

[9] Baala L, Romano S, Khaddour R, Saunier S, Smith UM, Audollent S (2007). The Meckel-Gruber syndrome gene, MKS3, is mutated in Joubert syndrome. Am J Hum Genet.

[10] Ramachandran U, Malla T, Joshi KS (2006). Meckel-Gruber syndrome. Kathmandu Univ Med J.

[11] de SilvaMV, Senanayake H, Siriwardana KD (2004). Meckel Gruber syndrome: occurrence in non-consanguineous marriages. Ceylon Med J.

[12] YucelU, Kayhanispahi C, Ayaz D, Bal F (2009). Early diagnosed meckel gruber syndrome. Perinatal J.

